# Misoprostol for primary versus secondary prevention of postpartum haemorrhage: a cluster‐randomised non‐inferiority community trial

**DOI:** 10.1111/1471-0528.13540

**Published:** 2015-09-01

**Authors:** S Raghavan, S Geller, S Miller, SS Goudar, H Anger, MC Yadavannavar, R Dabash, SR Bidri, MR Gudadinni, R Udgiri, AR Koch, MB Bellad, B Winikoff

**Affiliations:** ^1^ Gynuity Health ProjectsNew YorkNYUSA; ^2^ University of Illinois at ChicagoChicagoILUSA; ^3^ University of CaliforniaSan FranciscoCAUSA; ^4^ KLE University's Jawaharlal Nehru Medical CollegeBelgaumIndia; ^5^ BLDE University's Sri B. M. Patil Medical CollegeBijapurIndia

**Keywords:** Misoprostol, postpartum haemorrhage, primary prevention, secondary prevention

## Abstract

**Objective:**

To assess whether secondary prevention, which preemptively treats women with above‐average postpartum bleeding, is non‐inferior to universal prophylaxis.

**Design:**

A cluster‐randomised non‐inferiority community trial.

**Setting:**

Health sub‐centres and home deliveries in the Bijapur district of Karnataka, India.

**Population:**

Women with low‐risk pregnancies who were eligible for delivery with an Auxiliary Nurse Midwife at home or sub‐centre and who consented to be part of the study.

**Methods:**

Auxiliary Nurse Midwifes were randomised to secondary prevention using 800 mcg sublingual misoprostol administered to women with postpartum blood loss ≥350 ml or to universal prophylaxis using 600 mcg oral misoprostol administered to all women during the third stage of labour.

**Main outcome measures:**

Postpartum haemoglobin ≤7.8 g/dl, mean postpartum blood loss and postpartum haemoglobin, postpartum haemorrhage rate, transfer to higher‐level facilities, acceptability and feasibility of the intervention.

**Results:**

Misoprostol was administered to 99.7% of women as primary prevention. In secondary prevention, 92 (4.7%) women had postpartum bleeding ≥350 ml, of which 90 (97.8%) received misoprostol. The proportion of women with postpartum haemoglobin ≤7.8 g/dl was 5.9 and 8.8% in secondary and primary prevention clusters, respectively [difference −2.9%, one‐sided 95% confidence interval (CI) <1.3%]. Postpartum transfer and haemorrhage rates were low (<1%) in both groups. Shivering was more common in primary prevention clusters (*P *= 0.013).

**Conclusion:**

Secondary prevention of postpartum haemorrhage with misoprostol is non‐inferior to universal prophylaxis based on the primary outcome of postpartum haemoglobin. Secondary prevention could be a good alternative to universal prophylaxis as it medicates fewer women and is an acceptable and feasible strategy at the community level.

**Tweetable abstract:**

Secondary prevention of postpartum haemorrhage with misoprostol is non‐inferior to universal prophylaxis.

## Tweetable abstract

Secondary prevention of postpartum haemorrhage with misoprostol is non‐inferior to universal prophylaxis.

## Introduction

Universal prophylaxis (primary prevention) lowers mean postpartum blood loss, which reduces the incidence of postpartum haemorrhage (PPH) (blood loss ≥500 ml within 24 hours after delivery^1^). However, administration of prophylactic uterotonics (e.g. oxytocin or misoprostol) during third stage labour does not eliminate the need for treatment for some women.^2^ It is unclear whether universal prophylaxis saves women's lives, but it is evident that routine uterotonic prophylaxis is not 100% effective in preventing PPH. Clinical trials demonstrate that 6–16% of women still lose >500 ml blood despite prophylaxis.^3^, ^4^, ^5^, ^6^ Programs focusing solely on universal prophylaxis fail to meet the needs of all women. Moreover, cost, logistics and supply‐chain burdens of universal prophylaxis programs create challenges for sustainability.

We investigated a new service delivery model, which ‘treats’ incipient PPH early by offering uterotonics to a subset of women with above‐normal postpartum blood loss. This ‘secondary prevention’ model has the potential to be a less expensive alternative to universal prophylaxis, and exposes fewer women to side effects.^2^ Additionally, this strategy could efficiently focus resources on those women in danger of experiencing PPH.

A secondary prevention model may be most advantageous at the community level, where PPH treatment options and transfer to higher level care are commonly inaccessible. Oxytocin, the gold standard for PPH prevention and treatment,^7^, ^8^ is less available in the community, given refrigeration requirements and parenteral administration.^9^ Misoprostol, a prostaglandin E1 analogue, is a heat‐stable tablet. A substantial body of evidence has demonstrated its effectiveness for the prevention and treatment of PPH.^10^ The current non‐inferiority misoprostol trial was designed to assess whether a secondary prevention strategy resulted in maternal outcomes no worse than universal prophylaxis outcomes.

## Methods

The cluster‐randomised trial was implemented from December 2011 to March 2014 in the Bijapur district of Karnataka, India, and included deliveries conducted by Auxiliary Nurse Midwives (ANMs) at health sub‐centres and women's homes. Health sub‐centres, which are the lowest health post level within the Indian Rural Health System, are commonly staffed by one ANM and consist of one or two rooms with a delivery table, basic first aid medical equipment, and no refrigeration capabilities. The unit of randomisation for the study was ANMs, and deliveries enrolled by each ANM constituted a cluster.

Auxiliary Nurse Midwifes informed women about the study during antenatal care and administered eligibility screening and informed consent during early labour. Exclusions included women at high risk (as per the guidelines of the Ministry of Health of India, e.g. high blood pressure, multiple gestations) or in active labour at the time of consent. Informed consent was documented via the woman's signature or thumbprint. Information on the delivery was collected by ANMs using a standardised data collection instrument.

Auxiliary Nurse Midwifes provided the standard of care during the second stage of labour. Postpartum blood loss was collected for all women in a calibrated blood collection drape (Brasss‐V Drapes, Excellent Fixable Drapes, Madurai, Tamil Nadu, India). ANMs monitored blood loss and recorded the level 1 hour after delivery.

Auxiliary Nurse Midwifes allocated to universal prophylaxis administered 600 mcg (three 200‐mcg tablets) oral misoprostol (Misoprost^®^, Cipla, Mumbai, India) to women within 5 minutes of birth and gave routine postpartum care (which could include uterine massage, controlled cord traction, and cord clamping). ANMs allocated to secondary prevention administered 800 mcg (four 200‐mcg tablets) sublingual misoprostol, the recommended dose for postpartum haemorrhage treatment,^11^, ^12^ only if blood loss reached ≥350 ml on the collection drape (reports show a blood loss of >350 ml represents the top quartile of women with measured postpartum blood loss).^13^ ANMs recorded postpartum care and side effects associated with misoprostol (shivering, fever, headache, nausea, vomiting, diarrhoea, abdominal pain/cramping, palpitations, and seizures).

Auxiliary Nurse Midwifes were trained to diagnose PPH if blood loss was 500 ml or more, although diagnosis could be made based on other clinical factors (e.g. uterine tone, woman's general condition). If postpartum haemorrhage was diagnosed, the standard of care was provided, including transfer to higher‐level care. Research staff visited all enrolled women 72 hours (±8 hours) after delivery to measure postpartum haemoglobin via a portable handheld device (HemoCue^®^, Ängelholm, Sweden) and to collect information on acceptability of the interventions (problems taking misoprostol, preferences for future deliveries and recommendation to family/friends).

Before the trial commenced, 51 ANMs from three sub‐districts were stratified by sub‐district and delivery volume and randomised to the primary or secondary prevention strategy. In August 2012, seven ANMs from two additional sub‐districts were added following the same stratification and randomisation rules. Randomisation was performed by Gynuity Health Projects using a computer‐generated random sequence within each stratum. There was no masking because the studied interventions required distinct approaches to postpartum haemorrhage care that made masking impractical and would have inhibited assessment of program feasibility.

The primary outcome for this trial was the proportion of women with a postpartum haemoglobin ≤7.8 g/dl. This cut‐off value was based on reports of a mean pre‐delivery haemoglobin of 9.8 g/dl among women in India.^14^, ^15^, ^16^ A drop to 7.8 g/dl would approximate a clinically relevant 2 g/dl decrease in pre‐ to post‐delivery haemoglobin and is below the value considered indicative of moderate to severe anaemia (9.0 g/dl).^17^ Secondary outcomes included mean blood loss, diagnosis of PPH, mean postpartum haemoglobin, transfer to higher‐level facilities, use of additional interventions for haemorrhage, cost of implementing each strategy (results to be published separately), and acceptability and feasibility.

Secondary prevention was considered non‐inferior to primary prevention if the proportion of women with postpartum haemoglobin ≤7.8 g/dl in secondary prevention clusters was no more than 7% higher than in primary prevention clusters (previous community‐based studies were estimated to be 13%).^4^, ^5^ To assess this outcome, and assuming a 1:1 randomisation and accounting for clustering effect (using an intracluster correlation coefficient of 0.05), a sample of 3000 deliveries was needed [*α* = 0.05 (one‐sided), 80% power].^18^

Outcomes were compared by intervention and tested for statistical significance using the chi‐square test (cluster‐adjusted) for categorical variables and mixed linear models for continuous variables. Risk differences (difference between proportions of women for each outcome in primary and secondary clusters) and associated 95% confidence intervals were calculated. For the primary outcome, a one‐sided confidence interval was calculated for evaluating non‐inferiority; all other confidence intervals are two‐sided. Between‐group comparisons for severity and tolerability of side effects were made using an adjusted Wilcoxon two‐sample test.^19^ Multivariate analysis for the primary outcome was performed via generalised estimating equations to control for place of delivery and uterotonic administration prior to delivery. Analyses were performed using SAS 9.3 (SAS Institute Inc., Cary, NC, USA).

The protocol was approved by the Health Ministry's Screening Committee at the Indian Council of Medical Research (New Delhi, India) and by Institutional Review Boards at Jawaharlal Nehru Medical College (Belgaum, India) and the University of Illinois at Chicago (Chicago, IL, USA). An independent Data Safety Monitoring Board reviewed the study when half the enrolment was achieved. The study is registered with ClinicalTrials.gov (#NCT01462422).

## Results

**Figure 1:**
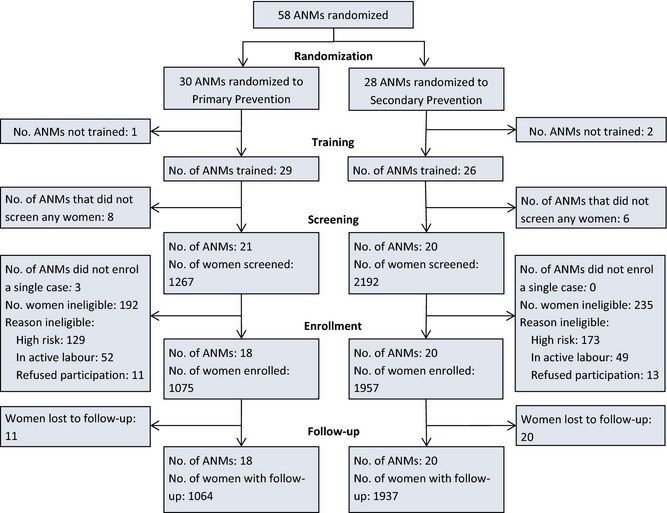
Trial profile.

Figure 1 shows the trial profile. In the primary prevention group, 1267 women were screened by 21 ANMs for study eligibility, of whom 1075 (84.8%) were enrolled by 18 ANMs (three ANMs screened but did not enrol any women). In the secondary prevention group, 20 ANMs screened 2192 women for study eligibility and enrolled 1957 (89.2%). The analysis of the primary outcome included 1064 women enrolled by 18 ANMs allocated to primary prevention and 1937 women enrolled by 20 ANMs allocated to secondary prevention. The trial stopped when the sample size of 3000 deliveries was achieved.

Baseline demographics and obstetric history were similar among women in primary and secondary prevention clusters (Table 1). Most deliveries occurred in sub‐centres (73–79%). Uterotonics were administered for labour augmentation before delivery in fewer than 10% of all deliveries.

**Table 1:** Baseline comparisons at the subject level

	Primary prevention	Secondary prevention
No. of clusters	18	20
No. of deliveries enrolled	1075	1957
**Demographics and obstetric history**		
Age, [*n*] mean (min,max)	[1071] 24.6 (18, 39)	[1956] 24.2 (17, 38)
# pregnancies, [*n*] mean (min,max)	[1072] 2.3 (0, 8)	[1956] 2.3 (1, 7)
Number of live births, [*n*] mean (min, max)	[1072] 1.2 (0, 7)	[1954] 1.3 (0, 6)
Estimated gestational age, [*n*] mean (min,max)	[1072] 38.1 (28, 44)	[1953] 37.7 (28, 44)
Known previous PPH, *n* (%)	9/1057 (0.9%)	11/1893 (0.6%)
**Delivery characteristics**		
Place of delivery, *n* (%)	*n *=1066	*n *= 1924
Sub‐centre	839 (78.7%)	1408 (73.2%)
Home	227 (21.3%)	516 (26.8%)
Uterotonic given before delivery, *n* (%)	92/1068 (8.6%)	189/1927 (9.8%)
Procedures performed during second or third stage of labour, *n* (%)	*n *=* *1064	*n *=* *1920
Uterine massage	735 (69.1%)	1385 (72.1%)
Controlled cord traction	551 (51.8%)	814 (42.4%)
Episiotomy	0 (0.0%)	3 (0.2%)

Few women (1.0% primary prevention, 1.8% secondary prevention) withdrew or were transferred to higher‐level care before delivery (prolonged labour in most cases). Of the remaining women, 99.7% in the primary prevention clusters received misoprostol according to the protocol. In secondary prevention clusters, 4.7% women had postpartum bleeding of ≥350 ml (compared with 1.9% in the universal prophylaxis clusters) and should have received misoprostol; 97.8% of these women received the intervention.

**Figure 2:**
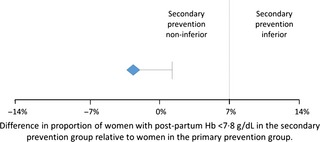
Non‐inferiority of secondary prevention relative to primary prevention. The diamond represents the point estimate of the difference in the primary outcome and the horizontal bar represents the one‐sided 95% CI for testing non‐inferiority. Non‐inferiority would be accepted if the 95% CI falls below the pre‐defined non‐inferiority margin of 7%.

The proportion of women with a postpartum haemoglobin measurement of ≤7.8 g/dl was 8.8 and 5.9% in primary and secondary clusters, respectively [risk difference −2.9%, upper bound of one‐sided 95% confidence interval (CI) 1.3%]. The difference and the 95% CI are below the non‐inferiority margin of 7.0% (Table 2, Figure 2). After controlling for place of delivery and uterotonic administration prior to delivery, the main effect was unchanged (*P* = 0.270, data not shown).

**Table 2:** Primary and secondary outcomes

	Primary prevention	Secondary prevention	ICC	Difference (SP‐PP)	
	(*n *=* *1075)	(*n *=* *1957)		Difference	95% CI of the difference^d^
**Primary outcome**					
Proportion with postpartum Hb ≤7.8 g/dl^a^	94/1064 (8.8%)	115/1937 (5.9%)	0.034	−2.9%	Up to 1.3%
**Non‐inferiority secondary outcomes**					
Rate of PPH^a^^,^ b	2/1064 (0.2%)	7/1920 (0.4%)	–	0.2%	–
Transfer to referral facilities for PPH^a^^,^ b	0/1064 (0.0%)	1/1920 (0.1%)	–	0.1%	–
**Other secondary outcomes**					
Blood loss (ml)^c^					
[*n*] mean (SD)	[1063] 173.9 (79.7)	[1915] 197.2 (78.9)	0.289	25.0	−10.3 to 60.4
Median (range)	175.0 (25, 525)	175.0 (25, 975)			
Additional uterotonic for treatment of suspected PPH at home/sub‐centre^b^	2 (0.2%)	2 (0.1%)	–	−0.1%	–
Postpartum Hb, [*n*] mean (SD)	[1064] 10.4 (1.9)	[1937] 10.6 (1.7)	0.027	0.14	−0.15 to 0.42

a Non‐inferiority measures.

b Rates are too small to allow valid confidence interval estimation and significance testing.

c Blood loss was not available for those subjects for whom a drape was not used. The mid‐point of each blood loss interval was used to estimate blood loss.

d The confidence interval for the primary outcome, the proportion of subjects with postpartum Hb ≤7.8 g/dL is one‐sided. All other confidence intervals are two‐sided.

PPH was diagnosed (based on clinical signs or blood loss >500 ml) in 0.2% (2/1064) and 0.4% (7/1920) of women in primary and secondary prevention clusters, respectively (Table 2). Additional uterotonics were administered for <0.5% of women in both clusters. Rates for PPH and additional uterotonics were too small to allow for valid confidence interval estimation.

**Figure 3:**
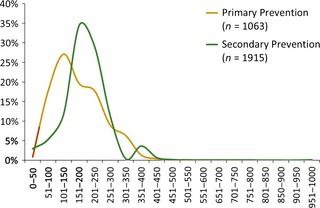
Postpartum blood loss.

Mean postpartum blood loss was lower in primary prevention clusters (173.9 ml, SD 79.7) than in secondary prevention clusters (197.2 ml, SD 78.9) but was not statistically significant (difference = 25.0 ml, 95% CI −10.3 to 60.4). There were no cases of severe PPH (blood loss >1000 ml). Figure 3 shows the distribution of measured blood loss by intervention arm.

One woman (0.1%) in secondary prevention clusters who received a blood transfusion at the referral centre was transferred to higher‐level care due to PPH (rates too small for valid significance testing, Table 2). Three women were transferred to higher‐level care after delivery for other reasons: one for severe shivering (primary prevention); one for retained placenta (secondary prevention); and one woman was transferred by the ANM for multiple gestation (secondary prevention).

Significantly more women in primary than secondary prevention experienced shivering after delivery (39.5 versus 9.0%, difference = −30.5, 95% CI −56.4 to −4.5; Table 3). The occurrence of other side effects did not differ by intervention. The majority of side effects were mild, with only 4.2 and 2.2% of women reporting moderate or severe shivering in primary and secondary groups, respectively (*P* = 0.151). Less than 1% of women in both groups described having ‘intolerable’ side effects.

**Table 3:** Side effects after delivery

	Primary prevention (%)	Secondary prevention (%)	ICC	*P*‐value
**Side effects after delivery of the baby**	**N = 1064**	**N = 1920**		
Shivering	420 (39.5)	173 (9.0)	0.402	0.013
Fever	5 (0.5)	7 (0.4)	0.005	0.746
Headache	25 (2.4)	63 (3.3)	0.137	0.762
Nausea	14 (1.3)	8 (0.4)	0.034	0.275
Vomiting	14 (1.3)	18 (0.9)	0.056	0.760
Diarrhoea^a^	0 (0.0)	1 (0.1)	—	—
Abdominal pain/ uterine cramping	64 (6.0)	328 (17.1)	0.399	0.285
Seizures	0 (0.0)	0 (0.0)	—	—
Palpitations^a^	1 (0.1)	0 (0.0)	—	—
**Severity of side effects and tolerability**				
*Severity of side effects – Shivering*	*N = 1064*	*N = 1920*		*P‐value* b
None	644 (60.5)	1747 (91.0)	0.378	0.151
Mild	376 (35.3)	131 (6.8)		
Moderate	35 (3.3)	34 (1.8)		
Severe	9 (0.9)	8 (0.4)		
*Severity of side effects – Headache*	*N = 1064*	*N = 1920*		
None	1039 (97.7)	1857 (96.7)	0.106	0.274
Mild	23 (2.2)	47 (2.5)		
Moderate	2 (0.2)	16 (0.8)		
Severe	0 (0.0)	0 (0.0)		
*Side effects – Tolerability*	*N = 1060*	*N = 1919*		
No side effects	578 (54.5)	1362 (71.0)	0.429	0.198
Tolerable	469 (44.3)	551 (28.7)		
Neutral	8 (0.8)	0 (0.0)		
Intolerable	5 (0.5)	6 (0.3)		

a Rates are too small to allow valid confidence interval estimation and significance testing.

b *P*‐values are two‐sided. For shivering severity, headache severity, and side effect tolerability, which are ordinal variables, counts and percentages are presented and *P*‐values are based on a Wilcoxon two sample test which accounts for clustering.^19^

Adherence to the intervention protocol was high in both sets of clusters (>99%). In only one case (<1%) an ANM in a secondary prevention cluster administered misoprostol before delivery of the baby; there was no adverse effect.

Information on acceptability was collected through an exit interview with all women who received misoprostol. Slightly more women in primary prevention reported no problem taking the pills (97.2 compared with 88.6% who took misoprostol sublingually). Most women in both clusters who received misoprostol said they would be willing to take the pills for future deliveries (99.3 versus 100%, respectively) and would recommend misoprostol to others (99.2 versus 98.9%, respectively).

## Discussion

### Main findings

This cluster‐randomised community trial shows that secondary prevention of PPH with misoprostol is non‐inferior to universal prophylaxis based on the primary outcome of postpartum haemoglobin. All outcomes in secondary prevention clusters, including rate of PPH and transfer, were found to be no worse than in primary prevention clusters. The secondary prevention strategy medicated substantially fewer women (5 versus 99%), who experienced significantly fewer side effects. Both strategies were feasible for implementation by ANMs at the community level.

This study showed an acute PPH rate of <0.5%, which is lower than prior published rates for women receiving misoprostol prophylaxis (6–16%).^3^, ^4^, ^5^, ^20^ In addition, approximately 5% of women bled ≥350 ml in secondary prevention clusters, which is lower than the 25% hypothesised based on prior studies.^13^ The reasons for the low levels of bleeding in this study are unclear, although other studies document low or declining measured ‘PPH rates’ during study recruitment, thought to be due to increased provider confidence and training.^21^, ^22^, ^23^, ^24^, ^25^ In compliance with government policy, ANMs referred high‐risk deliveries to higher‐level facilities, and women enrolled may have constituted a low‐risk population, although numerous studies have shown that it is difficult to identify the majority of women who will experience PPH based on pre‐existing risk factors. Regardless, medicating 5–25% of all women may be a practical and reasonable approach to managing PPH compared with universal prophylaxis, as PPH is not fully preventable.

Figure 3 compares blood loss curves among women in primary and secondary prevention clusters. The primary prevention strategy shifts the curve to lower levels of bleeding, but the reduction is found at <500 ml. The curves are almost identical at the 500‐ml mark and non‐existent at the 1000‐ml mark. These findings suggest that secondary prevention is no different from primary prevention for clinically meaningful bleeding. There is a small peak in the curve at 350–400 ml in the secondary prevention group which represents the point of misoprostol administration; this presumably reflects an eagerness among providers to intervene early by offering misoprostol to women who approach 350 ml blood loss.

### Strengths

The trial was implemented following a rigorous non‐inferiority trial design and included careful stratification of the clusters before randomisation. Consistent and strict study monitoring revealed that there was high compliance among ANMs in adhering to the protocol. The measurement of post‐delivery haemoglobin and postpartum blood loss via quantitative methods provided a systematic way to compare outcomes between the universal prophylaxis and secondary prevention groups.

### Limitations

During the course of the 2‐year trial, the Indian government's initiative to shift home and sub‐centre deliveries to higher‐level institutions impacted the pace of study recruitment. Some randomised ANMs did not deliver any women at home or sub‐centres, and we added new ANMs to our trial. Despite these efforts, more women were enrolled in the secondary prevention clusters compared with the universal prophylaxis clusters (1957 versus 1075). Careful monitoring revealed this difference to be due to chance. We have no reason to believe that there was any bias or selective recruitment or participation of women in the study. The proportion of screened women who enrolled in the study in both sets of clusters was similar (85% in the universal prophylaxis clusters and 89% in the secondary prevention clusters).

Also, due to logistical challenges, we could not collect pre‐delivery haemoglobin for enrolled women. Our primary outcome was based on published data from India on mean pre‐delivery haemoglobin^14^, ^15^, ^16^ and a cut‐off value that would reflect a clinically significant 2 g/dl average drop. We believe our assumption was reasonable and that the comparison of baseline characteristics indicates that clusters were homogeneous.

### Interpretation

This study shows secondary prevention to be a feasible strategy, although its implementation necessitates community level providers to identify women with above‐average bleeding. Birth attendants are in any case expected to identify and initiate transfers to higher‐level care for women with excessive bleeding. Secondary prevention would empower providers to offer misoprostol earlier, rather than waiting for late signs of haemorrhage or hypovolaemic shock. Providers can use methods such as a blood loss estimation tool or clinical signs, or some combination of these to identify women with above average bleeding. The 350‐ml marker used in this study is somewhat arbitrary and prompts providers to intervene for women with above average blood loss. New studies to evaluate alternative blood assessment tools (recognising that precision is not essential) such as blood mats or cloths could also help ‘trigger’ early treatment of PPH.

## Conclusion

Secondary prevention is an important step towards a more strategic (and potentially more cost‐effective and sustainable) placement of misoprostol for managing PPH along the continuum of care. As traditional definitions of prevention and treatment blur, this strategy offers policymakers a feasible and practical approach to address PPH at the community level.

## Disclosure of interests

None declared. Completed disclosure of interests form available to view online as supporting information.

## Contribution to authorship

SR was involved in the study design, development of research materials, monitoring of the clinical trial, interpretation of data, and manuscript writing. SR was involved in the study design, development of research materials, monitoring of the clinical trial, interpretation of data, and manuscript writing. SG, SM and SSG helped conceptualise the study, developed the trial design, protocol and study materials, monitored the clinical trial, interpreted the data, and wrote the manuscript. HA helped develop research materials, and was involved in study monitoring, data analysis and interpretation, and manuscript writing. ARK helped develop the protocol and study materials, conduct the data analysis and write the manuscript. MBB helped develop the protocol and study materials, monitor the trial and review the manuscript. MMY, RU, SRB, MRG were involved in the development of research materials, implementation and monitoring of the clinical trial, and manuscript review. RD and BW helped conceptualise the study, develop the protocol and study materials, and write the manuscript.

## Details of ethics approval

The Institutional Review Board of the University of Illinois at Chicago, approved the protocol on 18 November 2010 and provided an updated approval on 5 September 2012. The Institutional Ethics Committee on Human Subjects Research at the Jawaharlal Nehru Medical College at KLE University, Belgaum, India, approved the protocol on 20 January 2011. The Health Ministry's Screening Committee at the Indian Council of Medical Research, New Delhi, India, approved the protocol on 23 August 2011.

## Funding

This study was funded by the Bill and Melinda Gates Foundation.
